# First Aid to the Injured
*"First Aid to the Injured."—Five Ambulance Lectures by Dr. Frederick Esmarch, Professor of Surgery at the University of Kiel, etc. Translated from the German by H. R. H. Princess Christian. Second edition. Smith, Elder, and Co., London.)


**Published:** 1888-12-22

**Authors:** 


### "First Aid to the Injured."*

Since the introduction of ambulance societies and ambulance classes, which may be regarded as indirectly the offspring of the volunteer movement, several books of the kind have been published. The present small *brochure* has been translated from the German by her Royal Highness Princess Christian, who states that it is "not in the least degree meant as a substitute for Dr. Shepherd's handbook; but, having personally attended the ladies' classes of the Windsor Centre of the St. John's Ambulance Association, I felt the want of a more detailed account of the work aimed at than was supplied by notes made at the time." The lectures which comprise the book were delivered at the Samaritan Society School, at Kiel, and, in forming the class, Dr. Esmarch states he "followed the example of the English Order of St. John of Jerusalem."

Before proceeding to explain how to give judicious help in injuries and sudden accidents, Dr. Esmarch introduces a short account of the structure and organisation of the human body, illustrating the bones by a plate of the human skeleton, and the circulation of the blood by a diagram of the heart and blood-vessels. It may be doubted, however, if the latter will afford information to anyone unacquainted with anatomy, although a very good diagram for anatomical and physiological works, from which it has evidently been taken. A similar remark applies to the plate entitled, "A View of the Nervous System," and to the observations on the sympathetic nervous system, on the structure of the lungs, and on water and urea. Such subjects cannot be rendered so that those who run may read, in a few pages of wide letter-press even if aided by plates.

The best part of the book is undoubtedly that which treats of injuries, and especially of injuries of the blood-vessels. The different kinds of bleeding are clearly defined as follows: If after an injury "the blood does not flow freely, but trickles gently from the wound, only small blood-vessels have been divided. When dark blood wells out in a steady stream, and when the flow is increased by pressure applied above the wound, then a large vein has been opened. When bright red blood spurts out of the wound forcibly and in jets, an artery has been wounded. Unimportant hæmorrhages may be arrested by pressure on the wound itself, or by pressing the sides of the wound against each other. Hæmorrhage from an injured vein is sometimes difficult to stop on account of the pressure of some tight article of dress (a garter, for instance) above the bleeding-point. On loosening this, slight pressure and elevation of the limb suffice to arrest the bleeding. Should, however, bright blood continue to flow . . . a large artery must have been wounded . . . and those around should endeavour to stop the flow of blood. The only efficacious way of doing this is by firm pressure on the wound itself, if small, or on the trunk of the artery above the wound." How the arterial trunks should be controlled is shown in a series of six plates, which are sufficiently clear and well drawn.

Similar praise cannot, however, be accorded to some other sections of the lecture on injuries. For example, we are told that "in concussion of the brain you have fainting, insensibility, vomiting," which, to say the least, is a very meagre, and not altogether a correct description of the state known as concussion. The fact that in concussion of the brain the patient may be roused is not noted. Yet it is this fact which in a great measure serves to distinguish concussion of the brain, or "brain-shake," from compression of the brain, or the condition arising when from an injury a piece of bone or other substance is driven down on the brain. The distinctions between concussion and compression should have been made plain, and might have been mentioned as follows: In concussion or brain-shake insensibility takes place immediately on receipt of injury; in compression it generally comes on more gradually. In concussion the breathing is feeble or sighing; in compression it is slow, laboured, or snoring. In concussion the pulse is feeble; in compression it is full and bounding. In concussion the pupils of the eyes are contracted; in compression one or both are dilated. In concussion the surface of the body is pale and cold; in compression warm and moist. In concussion the patient can be roused so as to answer a question; in compression he cannot be roused. In concussion there is retching or vomiting; in compression not.

Next there is a section on fractured or broken bones, and on dislocations or bones out of joint, and the symptoms and signs of each class of injury are clearly given. But here, again, we notice a very serious omission. There is no comparison drawn between the signs of a fracture near a joint, and a dislocation of the joint. Yet this information is very necessary. If Dr. Esmarch had stated something of the kind, as below, his book would have been more valuable: Dislocations are distinguished from fractures near joints—first, by the absence of "grating" on movement of the injured parts; secondly, a fractured bone is more freely movable than natural, a dislocated bone less so; thirdly, if a fractured bone is drawn into its proper place, it will return so soon as the "extension" or pulling is discontinued, but a dislocated bone brought into its proper place will remain there; fourthly, by measurement of the bone supposed to be broken, which, if fractured, will be shortened, while the dislocated bone is of the natural length. Comparisons of length may be made with the bone of the sound limb. Again, with regard to sprains. While the symptoms of sprains are described, they are not contrasted as they should be with the symptoms of dislocation; yet sprains of joints so often happen that such contrast is most desirable. Dr. Esmarch should have said that dislocations are to be distinguished from sprains by pressing the swollen part steadily and firmly. If it be a dislocation, the end of the bone is felt firm and hard, while the swelling caused by a sprain is soft and yielding. Also by the facts that neither lengthening nor shortening of the limb are caused by sprains, while natural motion of the part, although painful, is possible. Several of the observations and recommendations made in this work are, to say the least, somewhat curious. In the article on burns and scalds we are unnecessarily told that "burns are caused by the concentrated heat of fire," and that, "should anyone have fallen into a lime-kiln, or soap-lye, he should be drawn out as quickly as possible." In his remarks on suffocation, Dr. Esmarch observes: "When a man who has gone down into a pit becomes unconscious, it is a proof of the dangerous state of the air in the pit." In the remarks on poisons, it is stated: "Poisons are substances which, taken internally, destroy life." In the treatment of poisoning the reader is told to "get, if you can, a piece of gutta-percha tubing, an inch in circumference, and if the patient is not unconscious, make him swallow twenty to twenty-five inches of it—enough to reach the stomach. Then raise the free end above his head, and by means of a funnel pour as much water down as the stomach will receive, etc., etc." Now, we undertake to say that no one but a professional swallower of swords could perform this boa-constrictor's feat of gorging twenty-five inches of gutta-percha tubing one inch in circumference; and still less likely a person in a state of excitement from fear of death by poisoning. Of course the tube could be passed into the stomach by another person, who knew the way to do it. But of this Dr. Esmarch does not inform us. In our opinion, the proper domestic treatment of most kinds of poisoning is to make the patient vomit at once, either by a mustard emetic (if the person can swallow), or by tickling his throat with a feather if he cannot swallow. After vomiting, or immediately if vomiting cannot be induced, the antidote to the poison should, if obtainable, be administered; and until aid comes it may be recollected, as Dr. Esmarch advises, that acids and alkalies act as antidotes to and neutralise one another; therefore, if an acid has been swallowed, alkalies dissolved in water should be at once given, viz., soda, potash, magnesia, lime-water. If an alkali has been taken, then give acids, viz., vinegar, lemon juice, etc.

* "First Aid to the Injured."—Five Ambulance Lectures by Dr. Frederick Esmarch, Professor of Surgery at the University of Kiel, etc. Translated from the German by H.R.H. Princess Christian. Second edition. Smith, Elder, and Co., London.)

